# Stress-induced nuclear translocation of CDK5 suppresses neuronal death by downregulating ERK activation via VRK3 phosphorylation

**DOI:** 10.1038/srep28634

**Published:** 2016-06-27

**Authors:** Haengjin Song, Wanil Kim, Jung-Hyun Choi, Sung-Hoon Kim, Dohyun Lee, Choon-Ho Park, Sangjune Kim, Do-Yeon Kim, Kyong-Tai Kim

**Affiliations:** 1 Department of Life Sciences, Pohang University of Science and Technology, Pohang, Gyeongbuk, 37673, Republic of Korea; 2 Division of Integrative Biosciences and Biotechnology, Pohang University of Science and Technology, Pohang, Gyeongbuk, 37673, Republic of Korea; 3 Department of Pharmacology, School of Dentistry, Brain Science and Engineering Institute, Kyungpook National University, Daegu, Gyeongbuk, 41940, Republic of Korea

## Abstract

Although extracellular signal-related kinase 1/2 (ERK 1/2) activity is generally associated with cell survival, prolonged ERK activation induced by oxidative stress also mediates neuronal cell death. Here we report that oxidative stress-induced cyclin-dependent kinase 5 (CDK5) activation stimulates neuroprotective signaling via phosphorylation of vaccinia-related kinase 3 (VRK3) at Ser 108. The binding of vaccinia H1-related (VHR) phosphatase to phosphorylated VRK3 increased its affinity for phospho-ERK and subsequently downregulated ERK activation. Overexpression of VRK3 protected human neuroblastoma SH-SY5Y cells against hydrogen peroxide (H_2_O_2_)-induced apoptosis. However the CDK5 was unable to phosphorylate mutant VRK3, and thus the mutant forms of VRK3 could not attenuate apoptotic process. Suppression of CDK5 activity results in increase of ERK activation and elevation of proapoptotic protein Bak expression in mouse cortical neurons. Results from VRK3-deficient neurons were further confirmed the role of VRK3 phosphorylation in H_2_O_2_-evoked ERK regulation. Importantly, we showed an association between phospho-VRK3 levels and the progression of human Alzheimer’s disease (AD) and Parkinson’s disease (PD). Together our work reveals endogenous protective mechanism against oxidative stress-induced neuronal cell death and suggest VRK3 as a potential therapeutic target in neurodegenerative diseases.

Protein kinases are crucial components involved in the integration of signal transduction networks, and the signaling pathways of mitogen-activated protein kinases (MAPKs)/Ras-Raf-MEK-ERK, which controls various cell responses, such as proliferation, differentiation, and metabolism, is most well-known in mammalian cells^1^,^2^. A growing evidence suggests that extracellular signal-regulated kinase 1/2 (ERK 1/2) also mediates cell death depending on the stimulus, the cell type, and the subcellular localization^1^. The regulation of cell proliferation and apoptosis relies on the balance, extent, and duration of pro- versus anti-apoptotic signals transmitted by ERK 1/2^1^,^2^. Sustained ERK activation causes its nuclear translocation and contributes to neuronal cell death via transcriptional regulation of pro-apoptotic proteins^3^.

Modulation of ERK activity is controlled by phosphorylation. Vaccinia H1-related (VHR) phosphatase specifically dephosphorylates and inactivates ERK 1/2 in the nucleus^4^,^5^. Vaccinia-related kinase 3 (VRK3), a member of the VRK family, is widely expressed in human tissues and increases VHR phosphatase activity through a direct binding^6^. *In silico* sequence analysis reveals that VRK3 contains five potential cyclin-dependent kinase 5 (CDK5) phosphorylation sites, including the repeated motif (K/R)XSPQXT(K/R)^7^,^8^. CDK5, a highly conserved proline-directed Ser/Thr kinase, is predominantly activated in the neurons because the expression of its activators, p35 and p39, are restricted to neuronal tissue^9^,^10^. Besides its roles in regulating cell migration, actin and microtubule dynamics, axonal guidance, and synaptic plasticity, CDK5 also controls neuronal survival during development^11^. MAPK/ERK kinase 1 (MEK1), which acts downstream of Ras in the MAPK pathway, is a well-known target of CDK5^12^. However, the identity of CDK5 substrates that link between CDK5 and the MAPK signaling pathway remains largely unknown.

Reactive oxygen species, such as hydrogen peroxide (H_2_O_2_), are generated during normal metabolic processes^13^, and play crucial roles in cellular signaling cascades^14^. However, an imbalance between ROS production and antioxidant defense mechanisms induces oxidative stress^15^, leading to the increase in ERK activation^16^,^17^. Unusually sustained ERK activation and its nuclear localization contribute to neuronal cell death^3^. The MAPK signaling pathways have been implicated in AD pathogenesis through divergent mechanisms including induction of neuronal apoptosis^18^,^19^,^20^ and phosphorylation of amyloid precursor protein (APP) and tau, which influence the cleavage of APP to generate amyloid-β (Aβ) and the formation of neurofibrillary tangle (NFT)s, two hallmarks of AD^19^,^21^. In addition, α-synuclein, a major component of Lewy bodies which are the pathological hallmark of PD, binds to ERK2 and forms a triple complex with transcription factor Elk-1, exerting significant suppressive effect on Elk-1 phosphorylation which causes dysfunction of neurons and leads to neurodegeneration^22^. Moreover, neuronal apoptosis induced by dysfunction of MAPK signaling pathway also involves increased level of α-synuclein^23^.

The present study investigated endogenous protective mechanisms against oxidative stress-induced neuronal cell death. Here we found that CDK5-mediated VRK3 phosphorylation at Ser 108 suppresses H_2_O_2_-induced prolonged ERK activation and subsequent cell death via enhancement of ERK phosphatase VHR activity. The role of VRK3 phosphorylation in H_2_O_2_-evoked ERK regulation was further confirmed in VRK3-deficient neurons. In the brains of AD and PD patients, we also observed increased levels of VRK3 phosphorylation, suggesting that VRK3 phosphorylation apparently works to resist oxidative stress-induced neuronal death. Hence, we can conclude that phosphorylated VRK3 plays a neuroprotective role through regulation of protein activities that mediate cell death and stress resistance.

## Results

### CDK5/p35 complex interacts with and phosphorylates VRK3 at Ser 108

**Figure 1:**
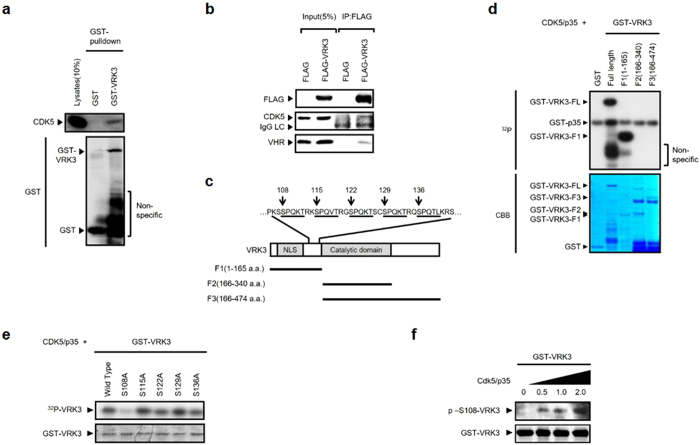
VRK3 is a substrate of CDK5. (**a**) CDK5/p35 interacts with VRK3 in a GST-pull down assay. (**b**) CDK5 immunoprecipitated with VRK3 in SH-SY5Y cell lysates expressing VRK3. (**c**) Putative CDK5 phosphorylation sites in the VRK3 sequence. Five proline-directed serine residues (↓) presented possible CDK5 action sites, including S108 (underlined). CDK5 recognition site is a repeated motif (K/R)*X*SPQ*X*T(K/R)^7^,^8^. (**d**) CDK5/p35 phosphorylates the F1 fragment that contained N-terminal region of VRK3 in an *in vitro* kinase assay. (**e**) S108 is a major phosphorylation site of VRK3 by CDK5. (**f**) Dose-dependent phosphorylation of VRK3 at S108 by CDK5/p35.

We first identified CDK5 as a binding partner of VRK3 by performing GST-pull down assay. GST-VRK3, but not GST control, specifically associated with endogenous CDK5 (Fig. 1a). Immunoprecipitation assay of SH-SY5Y cell extracts overexpressing flag-tagged VRK3 further confirmed the interaction between VRK3 and CDK5 (Fig. 1b). VHR, a known VRK3 binding partner, was also immunoprecipitated with CDK5 and VRK3. To assess whether VRK3 is phosphorylated by CDK5, and to identify the region of CDK5-mediated phosphorylation of VRK3, we performed an *in vitro* kinase assay by incubating GST-tagged recombinant full-length VRK3 and its fragments (F1, amino acids 1 to 165; F2, amino acids 166 to 340; F3, amino acids 166 to 474) with purified CDK5 complexed with its activator p35 (Fig. 1c). Consistent with previous results, co-incubation of VRK3 and CDK5 led to phosphorylation of VRK3, and CDK5/p35 complex robustly phosphorylated GST-VRK3-F1 (Fig. 1d). These data indicated that VRK3 is a substrate of CDK5 and phosphorylated in the N-terminal region, which contains five putative phosphorylation sites of CDK5 (Fig. 1c). To clarify phosphorylation sites of VRK3, we next conducted an *in vitro* CDK5 kinase assays using recombinant wild-type and mutants VRK3 (S108A, S115A, S122A, S129A, and S136A) in which Ala (A) replaced the potential phosphorylation sites (Pro-directed Ser 108, Ser 115, Ser 122, Ser 129 or Ser 136). CDK5/p35 complex phosphorylation was markedly attenuated in the GST-VRK3^S108A^ (Fig. 1e), indicating that CDK5 predominantly phosphorylates VRK3 at Ser 108.

To confirm Ser 108 as the site of CDK5/p35 complex-mediated VRK3 phosphorylation, we raised a phospho-specific VRK3 antibody with a synthetic phosphopeptide corresponding to VRK3 residues around Ser 108. This phospho-VRK3 antibody specifically recognized wild-type VRK3 when it was phosphorylated by CDK5 at Ser 108 and a phosphomimetic mutant of Ser 108 (GST-VRK3^S108E^) but did not recognize the S108A mutant (Supplementary Fig. S1a–e). The phosphorylation signal in the wild-type VRK3 was blocked by pre-incubation with Ser 108 phosphopeptide antigen (Supplementary Fig. S1f), suggesting that antibodies specific to the phosphorylated Ser 108 peptide were generated. We examined the expression of phosphorylated VRK3 using a phospho-specific antibody. CDK5/p35-mediated VRK3 phosphorylation on Ser 108 was increased in a concentration-dependent manner (Fig. 1f), confirming that VRK3 is a substrate of CDK5 and was phosphorylated at Ser 108.

### CDK5-mediated VRK3 phosphorylation enhances VHR phosphatase activity

**Figure 2:**
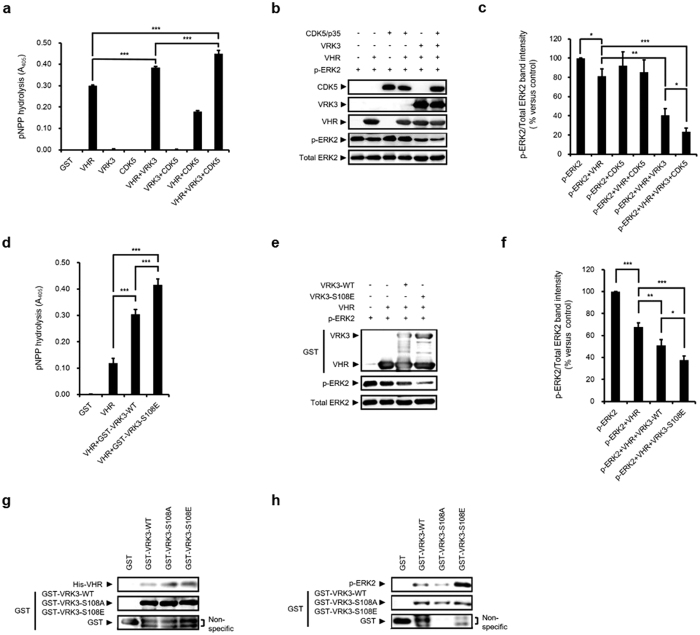
CDK5-mediated Ser108 phosphorylation of VRK3 enhances VHR activity. (**a**) CDK5/p35-mediated phosphorylation of VRK3 increases VHR phosphatase activity in pNPP assay. Values are normalized to GST-only controls, and shown as mean ± standard error of the mean (s.e.m.), *n* = 9. ***P < 0.001, ANOVA with post hoc Tukey’s test. (**b,c**) VRK3 phosphorylation by CDK5 enhances VHR phosphatase activity. *In vitro* VHR phosphatase assay performed using CDK5/p35 with recombinant p-ERK2. Quantification of the percentage of p-ERK2 versus total ERK2. Values are normalized to p-ERK2 expression, and shown as mean ± s.e.m., *n* = 5. *P < 0.05, **P < 0.01, ***P < 0.001, Student t-test. (**d**) S108 to glutamic acid (S108E) mutant VRK3 increases VHR phosphatase activity in pNPP assay. Values are normalized to GST-only controls, and shown as mean ± s.e.m., *n* = 8. ***P < 0.001, ANOVA with post hoc Tukey’s test. (**e,f**) Phosphomimetic mutant of VRK3 (S108E) enhances VHR phosphatase activity. *In vitro* VHR phosphatase assay performed using S108E with p-ERK2. Quantification of the percentage of p-ERK2 versus total ERK2. Values are normalized to p-ERK2 expression, and shown as mean ± s.e.m., *n* = 5. *P < 0.05, **P < 0.01, ***P < 0.001, Student t-test. (**g**) S108A or S108E mutant VRK3 has a negligible effect on the VHR-binding property of VRK3. (**h**) S108E, but not S108A mutant VRK3 promotes VRK3 binding to p-ERK2.

Our previous study demonstrated that direct binding of VRK3 to VHR increases its phosphatase activity, which specifically dephosphorylates and inactivates ERK in the nucleus^6^. To determine whether CDK5-mediated VRK3 phosphorylation affects VHR activity, we performed an *in vitro* phosphatase assay. Compared with VRK3 alone, the coexistence of active CDK5 and VRK3 increased phosphatase activity of VHR when either p-nitrophenyl phosphate (pNPP; Fig. 2a) or purified recombinant phospho-ERK2 protein (Fig. 2b,c) was used as a substrate. We replaced the CDK5/p35 complex with GST-VRK3^S108E^ in both the pNPP (Fig. 2d) and recombinant phospho-ERK2 protein assays (Fig. 2e,f). The addition of phosphomimetic mutant GST-VRK3^S108E^ induced an increase in VHR phosphatase activity when pNPP was used as a substrate (Fig. 2d). Consistently, VHR phosphatase activity was remarkably increased when purified recombinant phospho-ERK2 protein was used as a substrate in the presence of GST-VRK3^S108E^ (Fig. 2e,f). These results indicate that VRK3 phosphorylation enhances VHR phosphatase activity.

A number of studies have demonstrated that the regulation of the Raf-MEK-ERK cascade involves scaffolding proteins, such as kinase suppressor of Ras1 (KSR1), MEK partner-1 (MP1), and Sef^24^. To elucidate the mechanism of enhanced VHR phosphatase activity after VRK3 phosphorylation, we performed a VRK3 binding assay using recombinant VHR (Fig. 2g) or recombinant phospho-ERK2 (Fig. 2h). In the GST-pull down assay using recombinant proteins, the VHR binding was not different between the mutants and the wild-type VRK3, suggesting that VRK3 phosphorylation at Ser 108 does not affect VHR binding. The binding affinity of phospho-ERK2 to VRK3 was dramatically increased in VRK3^S108E^ compared to VRK3^WT^, indicating that Ser 108 phosphorylation of VRK3 increased binding affinity to the phospho-ERK2 protein. Together, these findings suggest that CDK5-mediated phosphorylation of VRK3 enhances VHR phosphatase activity by facilitating the recruitment of phospho-ERK to VHR.

### H_2_O_2_-induced activation of nuclear CDK5 leads to VRK3 phosphorylation and results in suppression of ERK activation

**Figure 3:**
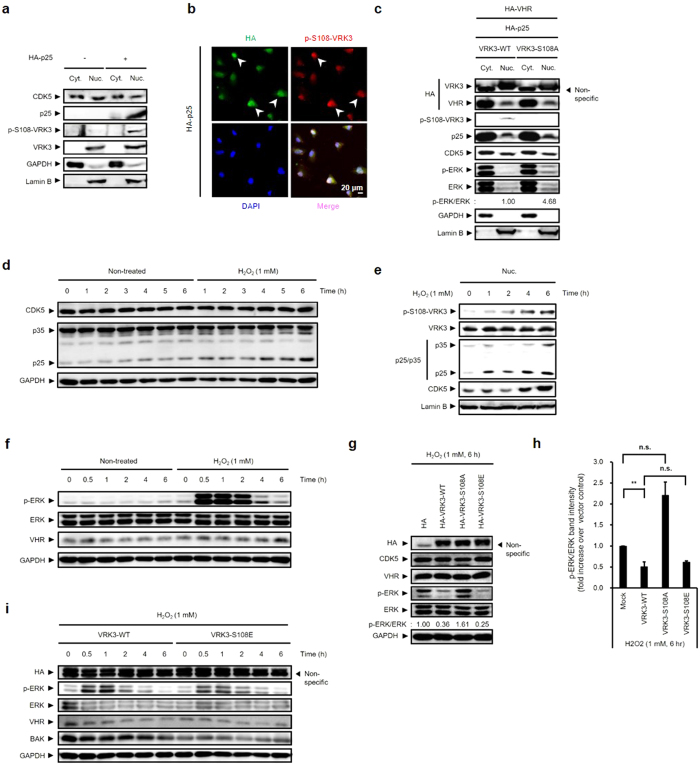
H_2_O_2_-induced activation of nuclear CDK5 leads to VRK3 phosphorylation and results in suppression of ERK activation. (**a**) Nuclear expression of p25 causes S108 phosphorylation of VRK3. (**b**) Cells expressing HA-p25 showed markedly increased VRK3 phosphorylation. White arrowheads highlight cells expressed relatively high level of HA-p25. Scale bar, 20 μm. (**c**) p25/CDK5-mediated VRK3 phosphorylation at Ser 108 is required for decreasing ERK activity in the nucleus. (**d**) H_2_O_2_ triggers the proteolytic cleavage of p35 to p25 in a time-dependent manner. (**e**) Increase in VRK3 phosphorylation at Ser 108 followed by the H_2_O_2_-induced proteolytic cleavage of p35 to p25 and nuclear translocation of CDK5 and its activators, p25 and p35. (**f**) H_2_O_2_ induces ERK activation in SH-SY5Y cells. (**g,h**) VRK3 phosphorylation at Ser 108 is required for the regulation of H_2_O_2_-induced ERK activation. Quantification of the percentage of p-ERK2 versus total ERK2. Values are normalized to p-ERK2 expression, and shown as mean ± s.e.m., *n* = 5. **P < 0.01, n.s. not significant. ANOVA with post hoc Tukey’s test. (**i**) Mimicking VRK3 phosphorylation suppresses H_2_O_2_-induced ERK activation in early time.

Nuclear CDK5 activity is necessary for VRK3 phosphorylation at Ser 108 because VRK3, which has a nuclear localization signal (NLS), is predominantly expressed in the nucleus. Although it is a widely accepted notion that a major CDK5 activator p35 is membrane-associated and mainly localized in cytoplasm due to a conserved N-terminal glycine myristoylation^25^,^26^, recent studies show that p35 displays a dynamic localization between the cytoplasm and the nucleus^27^, and nonmyristoylated p35 and p39 are preferentially accumulated in the nucleus^26^. In addition, p25, which lacks the myristoylation signal motif present at the N-terminus of p35, is a well-known nuclear activator of CDK5^11^. To examine whether nuclear CDK5 activity is enough to phosphorylate VRK3 at Ser 108, we tested cytoplasmic and nucleoplasmic fractions of SH-SY5Y cells expressing mock control or HA-p25 (Fig. 3a). VRK3 phosphorylation at Ser 108 occurred in the nucleoplasm, when p25 is overexpressed. Moreover, cells expressing HA-p25 exhibited markedly increased VRK3 phosphorylation (Fig. 3b). To confirm p25/CDK5-mediated VRK3 phosphorylation is important for VRK3-mediated ERK regulation in the nucleus, we analyzed cytoplasmic and nucleoplasmic fractions of SH-SY5Y cells (Fig. 3c). Although the nucleoplasmic fraction of SH-SY5Y cells exhibited relatively low levels of HA-p25 expression, it is sufficient to lead to VRK3 phosphorylation at Ser 108 in cells expressing VRK3^WT^. In the absence of stimulation that induces its nuclear translocation, ERK 1/2 are predominantly localized in the cytoplasm due to their interaction with anchors or scaffold proteins^28^. We showed that cells expressing VRK3^S108A^, a VRK3 mutant unable to be phosphorylated by CDK5/p25, exhibited sustained nuclear ERK activity compared with VRK3^WT^, even though the nuclear expression of ERK 1/2 was much lower than cytoplasm. Collectively, these observations indicate that nuclear CDK5 activity that contributes to Ser 108 phosphorylation of VRK3 is crucial for the reduced ERK activity in the nucleus.

Previous studies have shown that H_2_O_2_ stimulates cleavage of p35 to p25^29^ and increases ERK activity^17^, which subsequently results in oxidative stress-induced cell death^3^,^30^. To explore the physiological significance of CDK5-mediated VRK3 phosphorylation at Ser 108, we investigated H_2_O_2_-treated SH-SY5Y cells. Treatment with H_2_O_2_ stimulated cleavage of p35 to p25 in a time-dependent manner (Fig. 3d), and Ser 108 phosphorylation of VRK3 paralleled the nuclear translocation of CDK5 and p25 (Fig. 3e). Interestingly, consistent with previous studies that showed nuclear localization of p35^26^,^27^, obvious nuclear translocation of p35 was also detected about 6 h after H_2_O_2_ exposure.

ERK activation is also induced by H_2_O_2_ exposure (Fig. 3f). An increase in ERK phosphorylation reached a peak about 1 h, and returned to the baseline level about 6 h after H_2_O_2_ treatment. To clarify VRK3 phosphorylation in the regulation of oxidative stress-induced ERK activation, we examined H_2_O_2_-treated SH-SY5Y cells expressing wild-type or mutant VRK3 proteins (Fig. 3g,h). Consistent with our findings, overexpression of VRK3^WT^ downregulated p-ERK level compared with mock control and VRK3^S108A^. Contrary to our expectations, however, ERK activation after 6 h of H_2_O_2_ treatment was not different in cells expressing either VRK3^WT^ or VRK3^S108E^, a phosphomimetic mutant of VRK3. We analyzed SH-SY5Y cells expressing either VRK3^WT^ or VRK3^S108E^ after H_2_O_2_ exposure over a detailed time course to ascertain the involvement of VRK3 phosphorylation in the reduction of H_2_O_2_-induced ERK activation and the kinetics of oxidative stress response following H_2_O_2_ treatment (Fig. 3i). Higher level of p-ERK is detected in cells expressing VRK3^WT^ compared to VRK3^S108E^ until 1 h after H_2_O_2_ stimulation. However, the reduction in ERK activation in both cells was similar at the later time points of H_2_O_2_ treatment. In addition, the expression levels of Bak, a proapoptotic marker, was significantly elevated in cells expressing VRK3^WT^ compared to VRK3^S108E^ in early time of H_2_O_2_ treatment, and decrease thereafter. Taken together, H_2_O_2_ induced the cleavage of p35 to p25 as well as the nuclear translocation of CDK5 and its activators, p35 and p25, leading to VRK3 phosphorylation at Ser 108, which is responsible for the facilitation of VHR phosphatase activity and the decrease in phospho-ERK in the nucleus.

### VRK3 is required for the decrease in H_2_O_2_-induced apoptosis in SH-SY5Y cells

ERK activity is involved in neuronal development, neuronal plasticity, memory formation, and survival^31^,^32^. However, recent studies indicate that ERK activity mediates different antiproliferative events, such as apoptosis, autophagy, and senescence, depending on the duration and magnitude of activity, as well as its subcellular localization^1^,^2^. A wide body of evidence supports a detrimental role of persistent ERK signaling in neuronal response to stress^20^,^33^,^34^,^35^. Furthermore, unusually prolonged ERK activation has been implicated in oxidative stress-induced cell death^3^,^16^,^17^.

**Figure 4:**
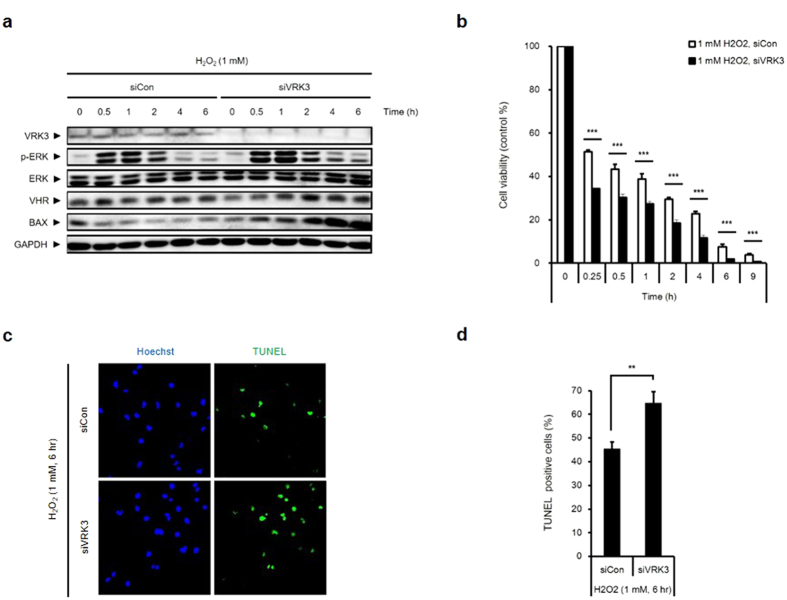
Protective action of VRK3 against H_2_O_2_-induced apoptosis in SH-SY5Y cells. (**a**) H_2_O_2_-induced ERK activation is sustained in VRK3 knockdown cells. (**b**) VRK3 downregulation reduces the viability of H_2_O_2_-treated SH-SY5Y cells. Values are normalized to the viability of non-treated cells, and shown as mean ± s.e.m., *n* = 8. ***P < 0.001, Student t-test. (**c,d**) Knockdown of VRK3 increases sensitivity of SH-SY5Y cells to H_2_O_2_-induced apoptosis. Quantification of the percentage of TUNEL-positive cells. Values are shown as mean ± s.e.m., *n* = 5. **P < 0.01, Student t-test.

A previous study on the role of VRK3 examined its function in neuronal differentiation^6^; however, VRK3 may affect ERK-mediated cell death as a negative regulator of ERK. We analyzed SH-SY5Y cells with or without VRK3 knockdown after H_2_O_2_ exposure to ascertain the involvement of VRK3 in the reduction of H_2_O_2_-induced ERK activation (Fig. 4a). The level of phospho-ERK was increased until 1 h after H_2_O_2_ treatment and decreased thereafter. Interestingly, reducing VRK3 expression using siRNAs show sustained phospho-ERK expression compared with control. In addition, the expression of Bax, a proapoptotic marker, increased in a time-dependent manner in VRK3 knockdown SH-SY5Y cells treated with H_2_O_2._

To confirm that VRK3 knockdown and subsequent sustained ERK activation in SH-SY5Y cells led to H_2_O_2_-induced cell death, we carried out the MTT and TUNEL assays to measure cell viability and apoptosis, respectively. Transient transfection of siVRK3 reduced the viability of H_2_O_2_-treated SH-SY5Y cells in a time-dependent manner (Fig. 4b). Furthermore, the number of TUNEL-positive cells markedly increased in VRK3-knockdown cells compared with control cells (Fig. 4c). Quantification of the signal showed up to 50% increase of TUNEL-positive cells, as compared with the level detected in the control cells (Fig. 4d). Taken together, these data indicated that VRK3 attenuates H_2_O_2_-induced apoptosis in neuroblastoma SH-SY5Y cells through suppression of prolonged ERK activation.

### Phosphorylation of VRK3 protects SH-SY5Y cells against H_2_O_2_-induced apoptosis

**Figure 5:**
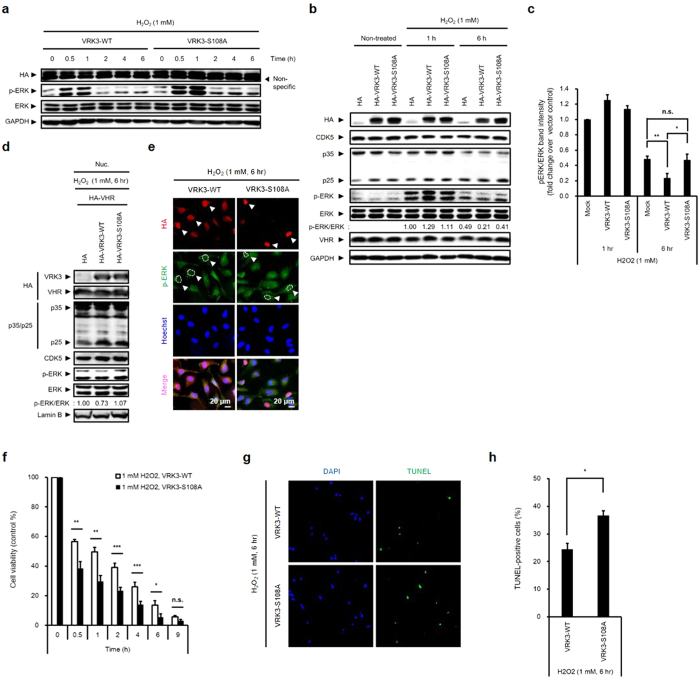
Phosphorylation of VRK3 protects cells against H_2_O_2_-induced apoptosis. (**a**) H_2_O_2_-induced ERK activation is sustained when VRK3 was not phosphorylated. (**b,c**) VRK3 phosphorylation at Ser 108 is important for the downregulation of H_2_O_2_-induced ERK activation. Quantification of p-ERK versus ERK western blot densitometry. Values are normalized to p-ERK level of the mock control, and shown as mean ± s.e.m., *n* = 4. *P < 0.05, **P < 0.01, n.s. not significant. ANOVA with post hoc Tukey’s test. (**d,e**) Reduction of H_2_O_2_-induced ERK activation in the nucleus is dependent on S108 phosphorylation of VRK3. White arrowheads highlight cells overexpressing wild-type or S108A mutant VRK3. Scale bar, 20 μm. (**f**) VRK3 phosphorylation at Ser 108 increases the viability of H_2_O_2_-treated SH-SY5Y cells. Values are normalized to the viability of non-treated cells, and shown as mean ± s.e.m., *n* = 8. *P < 0.05, **P < 0.01, ***P < 0.001, n.s. not significant. Student t-test. (**g,h**) S108A mutant VRK3 increases sensitivity of SH-SY5Y cells to H_2_O_2_-induced cell death. Quantification of the percentage of TUNEL-positive cells. Values are shown as mean ± s.e.m., *n* = 3. *P < 0.05, Student t-test.

To clarify whether Ser 108 phosphorylation of VRK3 affects regulation of H_2_O_2_-induced ERK activation in SH-SY5Y cells, we overexpressed VRK3^WT^ or VRK3^S108A^ (Fig. 5a). An increase in ERK phosphorylation was initially detectable at 30 min and reached a peak about 1 h after H_2_O_2_ treatment. The expression of active ERK in cells expressing VRK3^WT^ returned to the baseline level about 2 h after H_2_O_2_ administration. In contrast, although the protein expression levels of CDK5 and its activators, p35 and p25, were not different between the groups (Fig. 5b), ERK activity remained significantly higher than the basal levels after 6 h of H_2_O_2_ administration in cells overexpressing mock control and VRK3^S108A^ compared to VRK3^WT^-overexpressed cells (Fig. 5b,c).

As mentioned previously, sustained activation of ERK causes its translocation to the nucleus and promotes cell death^3^,^16^,^17^. To confirm whether CDK5-mediated VRK3 phosphorylation is required to suppress H_2_O_2_-induced active ERK in the nucleus, we examined the nucleoplasmic fraction of H_2_O_2_-treated SH-SY5Y cells (Fig. 5d). Nuclear ERK activation markedly reduced after 6 h of H_2_O_2_ administration in SH-SY5Y cells overexpressing VRK3^WT^ compared with cells expressing mock control and VRK3^S108A^. These observations are further supported by immunofluorescence (Fig. 5e). Collectively, CDK5-mediated VRK3 phosphorylation at Ser 108 played a significant role in the inhibition of H_2_O_2_-induced persistent ERK activation in the nucleus.

We next determined the relevance of Ser 108 phosphorylation of VRK3 in H_2_O_2_-induced apoptosis of SH-SY5Y human neuroblastoma cells. Overexpression of mutant VRK3 inhibited the viability of SH-SY5Y cells treated with H_2_O_2_ in a time-dependent manner (Fig. 5f). TUNEL staining also showed that overexpression of VRK3^S108A^ increased H_2_O_2_-induced apoptosis compared with VRK3^WT^ (Fig. 5g,h). Taken together, these results suggest that Ser 108 phosphorylation of VRK3 protected SH-SY5Y cells against H_2_O_2_-induced apoptosis through suppression of sustained ERK activation.

### Neuroprotective effect of phosphorylated VRK3 on H_2_O_2_-induced apoptosis in mouse cortical neurons

**Figure 6:**
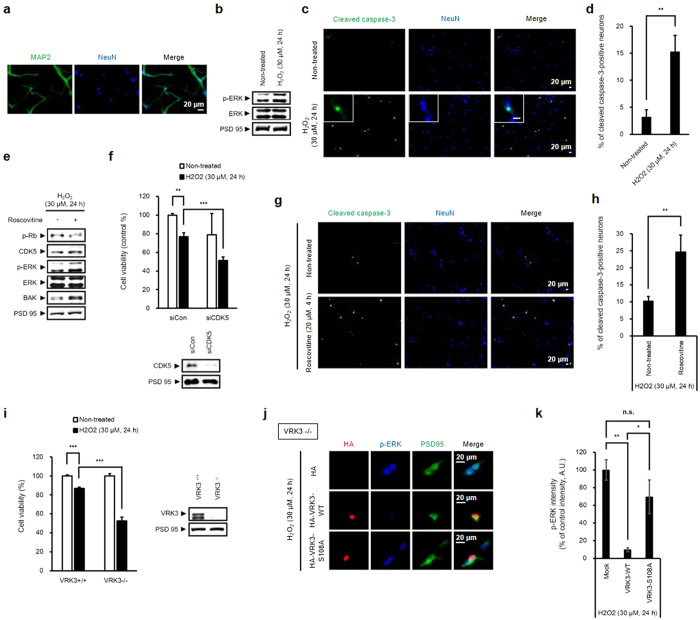
VRK3 phosphorylation is protective against H_2_O_2_-induced apoptosis in mouse cortical neurons. (**a**) Primary cultured mouse cortical neurons expresses MAP2 and NeuN. Scale bar, 20 μm. (**b**) H_2_O_2_ induces ERK activation in mouse cortical neurons. (**c,d**) H_2_O_2_ treatment causes apoptosis of mouse cortical neurons. Representative images show cleaved caspase-3-positive cells and NeuN-stained cell nuclei. Scale bar, 20 μm. Quantification of the percentage of cleaved caspase-3-positive cells. Values are shown as mean ± s.e.m., n = 3. **P < 0.01, ANOVA with post hoc Tukey’s test. (**e**) Downregulation of CDK5 activity leads to increase of H_2_O_2_-induced ERK activation and elevation of proapoptotic protein Bak expression. (**f**) CDK5-deficient neurons had an increased sensitivity to H_2_O_2_-induced cell death. Values are shown as mean ± s.e.m., n = 3. **P < 0.01, ***P < 0.001, ANOVA with post hoc Tukey’s test. (**g,h**) Suppression of CDK5 activity results in apoptosis of mouse cortical neurons. Representative images show cleaved caspase-3-positive cells and NeuN-stained cell nuclei. Scale bar, 20 μm. Quantification of the percentage of cleaved caspase-3-positive cells. Values are shown as mean ± s.e.m., n = 5. **P < 0.01, Student t-test. (**i**) VRK3-deficient mice shows increased sensitivity to H_2_O_2_-induced apoptosis. Values are shown as mean ± s.e.m., n = 3. ***P < 0.001, ANOVA with post hoc Tukey’s test. (**j,k**) Overexpression of wild-type VRK3, not mutant VRK3, downregulated H_2_O_2_-induced persistent ERK activation in VRK3-deficient neurons. Scale bar, 20 μm. Quantification of phospho-ERK in VRK3-deficient neurons expressing wild-type or S108A mutant VRK3. Values are normalized to HA-only controls in arbitrary units (A.U.), and shown as mean ± s.e.m., *n* ≥ 30 for each sample. *P < 0.05, **P < 0.01, n.s. not significant. Student t-test.

To determine whether VRK3 and its phosphorylation affect the regulation of nuclear ERK in mouse cortical neurons treated with H_2_O_2_, cortical neurons obtained at postnatal days 0.5 were purified and cultured for 5 days. Cultured cells were considered neurons based on the presence of MAP2- and NeuN-positive projections (Fig. 6a). H_2_O_2_-evoked ERK activation was also detected in mouse cortical neurons (Fig. 6b). In addition, H_2_O_2_ treatment significantly increased the number of cleaved caspase-3-positive cells (Fig. 6c,d), suggesting that H_2_O_2-_induced persistent ERK activation causes neuronal cell death.

To confirm the involvement of CDK5 in the regulation of oxidative stress-induced ERK activation, we examined mouse cortical neurons downregulating CDK5 activity by either CDK5 inhibitor roscovitine (Fig. 6e,g,h) or siRNA (Fig. 6f). Suppression of CDK5 results in increase of ERK activation and elevation of proapoptotic protein Bak expression (Fig. 6e). In addition, CDK5-deficient neurons showed markedly decreased cell viability after H_2_O_2_ treatment compared to control (Fig. 6f). Moreover, downregulation of CDK5 activity significantly increased the number of cleaved caspase-3-positive cells (Fig. 6g,h), indicating that CDK5 activity is required to protect neurons from H_2_O_2_-induced excessive ERK activation that triggers cell death.

To directly examine the functional importance of VRK3 *in vivo*, we generated mice deficient for VRK3 by targeted gene disruption in embryonic stem cells. VRK3-deficient neurons showed markedly decreased cell viability following H_2_O_2_ treatment compared with control (Fig. 6i). Results from immunocytochemistry using VRK3-deficient neurons revealed that these increased sensitivity to H_2_O_2_ treatment was due to sustained ERK activation (Fig. 6j,k). VRK3-deficient neurons overexpressing wild-type VRK3 exhibited dramatically reduced ERK activity compared with control which showed sustained ERK activation for at least 24 h after H_2_O_2_ exposure. In contrast, ERK activity remained higher after 24 h of H_2_O_2_ administration in VRK3- deficient neurons overexpressing mutant VRK3 (VRK3^S108A^), indicating that phosphorylation of VRK3 became more effective for maintaining neuronal viability against H_2_O_2_-evoked persistent ERK activation and subsequent cell death.

### Relevance to human Parkinson’s and Alzheimer’s disease patients

Oxidative stress, defined as the cellular imbalance between the generation and scavenging of ROS^15^, has been associated with cell damage and progressive cell death that occurs in neurodegenerative disorders such as AD and PD^36^,^37^. Several studies have shown abnormally elevated ERK activation and accumulation in the same vulnerable neurons in AD that exhibit oxidative damage and in pigmented substantia nigra neurons from PD^18^,^38^.

**Figure 7:**
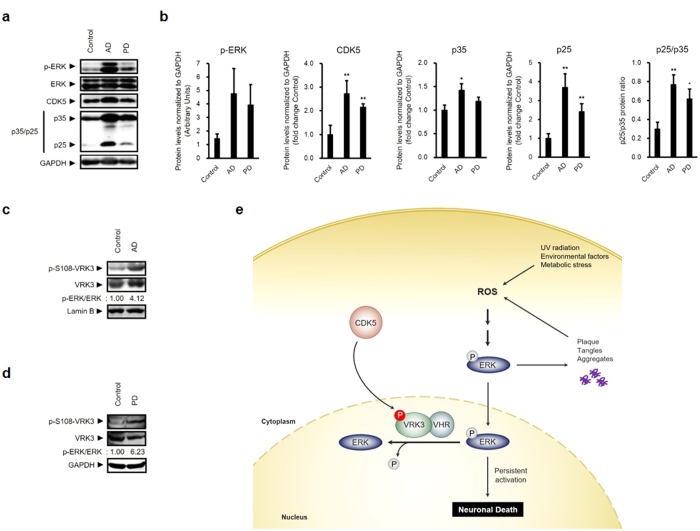
Phospho-VRK3 is increased in the brains of Alzheimer’s and Parkinson’s disease patients. (**a,b**) Phospho-ERK, CDK5, and its activators, p35 and p25, were increased in the prefrontal cortex (PFC) of AD and PD patients compared with non-demented control individuals. Quantification of phospho-ERK, CDK5, p35, p25 and p25/p35 ratio in AD (*n* = 4), PD (*n* = 4), and control (*n* = 4) brains. Values are normalized to GAPDH (**c,d**) VRK3 phosphorylation was increased in brain lysates from the same regions of the prefrontal cortex in AD and PD. (**e**) Proposed model for the protective role of CDK5 and VRK3 against neuronal death induced by oxidative stress. Oxidative stress causes prolonged ERK activation, which is responsible for the formation of Aβ plaque, NFTs, protein aggregates, and subsequent neuronal cell death. Activated CDK5 contributes to neuronal protection from oxidative stress-induced cell death through phosphorylation of VRK3 at S108, which enhances VHR phosphatase activity and negatively regulates sustained ERK activity in the nucleus.

To determine whether our findings have clinical relevance in these diseases, we analyzed brain lysates from AD and PD patients (Supplementary Table S1). Consistent with previous reports, the level of phospho-ERK is dramatically increased in the brains of AD and PD patients (Fig. 7a,b). In addition, elevated levels of CDK5 and its activators, p35 and p25, are detected in patients with AD and PD compared with normal brain control. In these patients VRK3 phosphorylation at Ser 108 is also markedly increased (Fig. 7c,d). Together, these findings suggest that phosphorylated VRK3-mediated protective signaling to resist the stress exists in the human brains of AD and PD.

## Discussion

Oxidative stress induces neuronal death through prolonged ERK activation. However, the endogenous protective mechanisms against oxidative stress-induced neuronal cell death, which is associated with several neurodegenerative diseases including AD and PD, remains largely unknown. Here we report that oxidative stress-induced CDK5 activation stimulates neuroprotective signaling via phosphorylation of VRK3, which plays an important role in negative feedback inhibition of persistent ERK activation (Fig. 7e).

Although CDK5 is located in both cytoplasm and nucleus^39^, most studies on CDK5 are focused on the cytoplasmic functions including the regulation of cytoskeletal dynamics, migration, and membrane trafficking. However, recent studies show that p35 could be localized in the nucleus^40^. It is only recently that the survival roles of nuclear CDK5 have been elucidated. Nuclear translocated CDK5 in respond to neuronal activity phosphorylates methyl-CpG-binding protein 2 (MeCP2), and regulates its transcriptional repressor activity which is critical for activity-dependent *bdnf* transcription and dendrite development^41^. The present study identified VRK3 as a novel nuclear substrate of CDK5. Oxidative stress-induced nuclear translocation of CDK5 alters its substrate specificity^11^ and mediates VRK3 phosphorylation at Ser 108 via p25/p35 binding-mediated activation. Phosphorylated VRK3 protects cells against H_2_O_2_-induced neuronal death through suppression of prolonged ERK activation.

The survival role of CDK5 is based, in part, on crosstalk interactions between death and survival signaling pathways. Although previous studies have shown that CDK5-mediated phosphorylation of MEK1, the upstream activator of ERK, represses transient activation induced by nerve growth factors in PC12 cells^42^, the relationship between CDK5 and MAP kinase pathway is still poorly understood. The present study demonstrated that oxidative stress-induced nuclear translocation of CDK5 promotes VRK3 phosphorylation, leading to inhibition of sustained ERK activation that triggers cell death. Phosphorylation of VRK3 by CDK5 seemed to act as a “molecular switch” that modulate the duration of ERK activation.

ERK is a hub protein kinase that signals many cellular factors from various receptors and elicits a variety of cellular responses. Duration of ERK activation and its subcellular localization are important because constitutively active ERK is translocated from cytoplasm to the nucleus and promotes neuronal cell death through transcriptional regulation of pro-apoptotic genes^3^. This is consistent with our present work that showed VRK3-deficient cells, which display prolonged ERK activation, exhibit prominent cell death following H_2_O_2_ treatment. In addition, overexpression of VRK3^S108A^, but not VRK3^WT^, had an increased sensitivity to H_2_O_2_-induced cell death. CDK5-mediated VRK3 phosphorylation have enhanced VHR phosphatase activity by facilitating the recruitment of phospho-ERK to VHR. VRK3 knockdown or mutation on the Ser 108 residue prolonged nuclear retention of activated ERK, which promotes cell death. It seems that VRK3 phosphorylation prevents prolonged nuclear ERK activation through downregulation of nuclear ERK activity by VHR. Moreover, the overexpression of VRK3^S108E^, a phosphomimetic VRK3 mutant, suppresses oxidative stress-induced ERK activation in early stage compared with VRK3^WT^. Relatively higher expression level of Bak in cells expressing VRK3^WT^ gradually decreased and reached similar levels to that of VRK3^S108E^. It appears that time-dependent generation and translocation of p25 and p35 to the nucleus after H_2_O_2_ treatment induces VRK3^WT^ phosphorylation and contributes to the reduction of ERK activation. Because the formation of A_β_ peptide and NFTs is associated with ERK-mediated phosphorylation of APP and tau^19^,^21^ and abnormal MAPK signaling pathway results in increase of α-synuclein^23^, enhancement of VHR phosphatase activity through VRK3 phosphorylation could protect neurons from neurodegeneration.

Under normal physiological conditions, VRK3 negatively regulates ERK activity along with VHR, causing timely and transient action of ERK. Oxidative stress leads to abnormal ERK activation and subsequently elicits activation of protective signaling to resist the stress. Stress conditions activate CDK5, which phosphorylates VRK3 at Ser 108 to increase VHR phosphatase activity, and suppress prolonged ERK activation that causes cell death. Furthermore, CDK5-mediated VRK3 phosphorylation at Ser 108 was elevated in post-mortem brain samples from patients with PD and AD. Therefore, VRK3 could be an attractive candidate to reduce neurodegeneration. It is likely that VRK3 has a protective role against oxidative stress in the early stages of neurodegenerative diseases. However, accumulation of oxidative damage in later disease stages may be overwhelming and cause death-promoting factors to reach a certain threshold, thereby triggering cell death. These findings are suggests a link to human neurodegenerative conditions and protective mechanisms against oxidative stress-induced neuronal death. Thus, VRK3 may be the foundation of a therapeutic strategy for neurodegenerative conditions.

## Methods

### Brain sample procurement

Post-mortem human brain tissue from control subjects, Alzheimer’s and Parkinson’s patients was obtained from the Netherlands Brain Bank (NBB) in accordance with institutional guidelines and approved protocols. The NBB only supplies material that has been obtained on the basis of informed consent of the brain donor. The samples analyzed in this study were strictly defined according to Braak and Braak staging^43^,^44^, with control samples being brains that were completely devoid of Lewy bodies and Aβ pathologies (i.e., Braak and Braak stage 0–2). The characteristics of these samples are provided in Supplementary Table S1.

### Protein extraction from human brain tissue

Snap-frozen post-mortem human brain tissue was ground and homogenized in Triton lysis buffer (20 mM Tris, 150 mM NaCl, 1 mM EDTA, 1 mM EGTA, 1% Triton-X, 2.5 mM sodium pyrophosphate, 1 mM β-glycerophosphate, 1 mM Na_3_VO_4_) supplemented with protease inhibitors. Samples were sonicated and centrifuged at 15,000 rpm for 30 min at 4 °C. The supernatant was removed, and the protein concentration was determined using Bradford Reagent (AMRESCO). Samples were denatured for 5 min at 95 °C with SDS sample buffer containing β-mercaptoethanol. Proteins were separated by SDS-PAGE and transferred to nitrocellulose membranes. Western blots were quantified by densitometry using ImageJ software.

### Mice

Specific pathogen-free (SPF) VRK3 knockout mice were bred and maintained on a C57BL/6 background. All animals were maintained on food and water ad libitum with 12-hr light-dark cycle. Approval of the study protocol was obtained from the Pohang University of Science and Technology Institutional Animal Care and Use Committee (POSTECH IACUC). All animal experiments were carried out according to the provisions of the Animal Welfare Act, PHS Animal Welfare Policy, and the principles of the NIH Guide for the Care and Use of Laboratory Animals. All mice were maintained under conventional conditions at the POSTECH animal facility under institutional guidelines.

### Primary neuron culture and transfection

Cortices were dissected from P0.5 newborn pups of wildtype and VRK3 knockout mice. Cultured cortical neurons were seeded onto a poly-L-lysine-coated culture dishes and were maintained in Neurobasal Medium supplemented with GlutaMAX, B27 supplements, and penicillin-streptomycin. Neuronal transfections were performed with Lipofectamine 2000 at 3 days *in vitro* (DIV) according to the manufacturer’s instructions.

### Antibodies

The following monoclonal antibodies were purchased: anti-p-ERK (abcam); anti-GAPDH (Santa Cruz Biotechnology); anti-VHR (BD Biosciences); anti-HA (Roche); anti-NeuN (Merck Millipore); anti-GST, anti-cleaved caspase-3, anti-Bax, and anti-Bak (Cell Signaling Technology). The following polyclonal antibodies were also obtained: anti-PSD95 (abcam); anti-VRK3, anti-VHR, anti-p-ERK, anti-ERK, and anti-His tag (Cell Signaling Technology); anti-VRK3 (Atlas antibodies); anti-HA epitope (YPYDVPDYA; Bethyl Laboratories); anti-FLAG epitope (D-8), anti-Lamin B (C-20), anti-MAP2 (I-18), anti-CDK5 epitope (C-8), and anti-p35 epitope (C-19) (Santa Cruz Biotechnology). Phos-VRK3 at Ser 108 (pS108) polyclonal antibody was generated and purified from two rabbitts and two rats immunized with carrier protein-conjugated phosphopeptide, GSRPPTPKSPQKTRKSPQ (residues 99–117 of human VRK3; AnyGen, Gwangju, Korea).

### Plasmid construction

The coding region of VRK3, VHR, and p25 were amplified by RT-PCR from the mRNA of SH-SY5Y, HEK293A, and HeLa cells and cloned into pFLAG-CMV2, pcDNA3.1-HA, pGEX4T-3, pEGFP-N1, or pProEX-Hta vectors. Partial fragments of VRK3 F1 (amino acids 1–165), F2 (amino acids 166–340), and F3 (amino acids 166–474), were cloned into pGEX4T-3 vector. VRK3 mutants (S108A, S115A, S122A, S129A, S136A and S108E) were generated by PCR-based site-directed mutagenesis (SDM) into pGEX4T-3 vector.

### Cell culture, transfection, and RNA interference

SH-SY5Y cells were grown in minimal essential medium (MEM) supplemented with 10% heat-inactivated fetal bovine serum (FBS) and 100 U/ml each of penicillin G and streptomycin. Transient transfections were performed by electroporation using a Microporator MP-100 (Digital Bio) or Neon-Transfection System (Invitrogen), and by lipid-mediated delivery using METAFECTENE (Biontex) according to the manufacturer’s instructions. Scrambled siRNA (5′-CCU ACG CCA CCA AUU UCG U(dTdT)-3′) and SMARTpool containing four pooled SMART-selected siCDK5 duplexes were obtained from Upstate Biotechnology (GE Healthcare, Dharmacon). VRK3 siRNA (5′-GGA CAA AUU GCC UUC CCA A(dTdT)-3′, Bioneer) targets the 1169–1187 bp of VRK3 mRNA.

### Purification of fusion proteins

All GST- or His-tagged fusion proteins were expressed in *Escherichia coli* BL21 (DE3) pLysS (Novagen) and purified using glutathione-sepharose 4B agarose beads (GE Healthcare Bio-Sciences) or Ni-NTA agarose (Invitrogen) according to the manufacturer’s instructions.

### Peptide competition assay (PCA)

Phosphospecific antibodies (1 μg/ml) was incubated for 2 h at 37 °C in 5% skim milk Tris-buffered saline (TBS) solution containing 0.1% Tween-20, with or without a 200-fold molar excess of peptide (200 μg), which was used as an antigen for antibody production. These solutions, which contained antigen-antibody complexes or antigen alone, were incubated with PVDF membrane-bound antigen (transferred proteins after *in vitro* kinase assay) overnight at 4 °C.

### *In vitro* binding assay

GST or GST-tagged proteins were incubated overnight with SH-SY5Y cell lysates or other recombinant proteins in Triton lysis buffer containing 1 mM DTT and 10% glycerol, followed by incubation with glutathione-sepharose 4B beads (GE Healthcare Bio-Sciences) at 4 °C for 1 h.

### Immunoprecipitation

Cells were washed with chilled phosphate-buffered saline (PBS) and lysed for 30 min on ice using Triton lysis buffer supplemented with protease inhibitors (Roche). Lysates were sonicated and clarified by centrifugation at 15,000 rpm for 30 min. Immunoprecipitation was performed by incubating antibodies with lysates overnight, followed by incubation with protein G beads (Roche) at 4 °C for 1 h.

### Kinase and phosphatase activity assay

For measuring the *in vitro* kinase activity of CDK5, purified proteins were incubated with active CDK5/p35 complex (Merck Millipore) in kinase reaction buffer (50 mM Tris at pH 7.5, 5 mM MgCl_2_, 0.5 mM DTT, 150 mM KCl, and [γ-^32^P]ATP for 30 min at 30 °C. For measuring the *in vitro* phosphatase activity of VHR, purified proteins were further incubated with 5 × 30 mM of the phosphatase substrate p-nitrophenol phosphatase hexahydrate (P4744, Sigma-Aldrich) dissolved in recommended buffer (1 M diethanolamine at pH 10.4 with 0.5 mM MgCl_2_) for 2 h at 37 °C or with recombinant p-ERK2 (gift from H.S. Yoon, Nanyang Technological University, Singapore) for 1 h at 37 °C.

### Preparation of cytoplasmic and nuclear extracts

To prepare cytoplasmic extracts, SH-SY5Y cells were lysed at 4 °C in a lysis/extraction buffer containing 10 mM HEPES (pH 7.6), 3 mM MgCl_2_, 40 mM KCl, 0.5% NP-40, 5% glycerol, 2 mM DTT, and 0.5 mM PMSF supplemented with protease inhibitors. After incubation on ice for 15 min, cellular debris was removed by centrifugation (3,000 rpm) at 4 °C for 5 min. Nuclei were resuspended and spun down twice in lysis/extraction buffer to avoid contamination of cytoplasmic proteins. To prepare nuclear extracts, pelleted nuclei were placed in nuclear extraction buffer containing 10 mM HEPES (pH 7.9), 0.1 mM EGTA, 1.5 mM MgCl_2_, 420 mM NaCl, 25% glycerol, 0.5 mM DTT, and 0.5 mM PMSF. After incubation on ice for 15 min, samples were sonicated and centrifuged (15,000 rpm) at 4 °C for 10 min. The supernatant was removed, and protein concentration was analyzed using Bradford reagent (AMERSCO).

### Assessment of cell viability and apoptosis

Cell viability was assessed using a chromogenic assay involving biological reduction of the tetrazolium salt 3-(4,5-dimethylthiazol-2-yl)-2,5-diphenyltetrazoliumbromide (MTT), which is converted into blue formazan crystals by living cells. After electroporation with DNA or siRNA, cells were subcultured in 96-well plates at a density of 1 × 10^4^ cells for 24 h in a final volume of 100 μl. Cells were then cultured in serum-free medium containing H_2_O_2_ for the indicated period of time. After the medium was removed, MTT (5 mg/ml in PBS) was added to the culture medium. Cells were incubated for 2 h in the dark at 37 °C, and the cell-free supernatant was removed from the wells. A total of MTT solvent (4 mM HCl and 0.1% Nondet P-40 [NP40], both in isopropanol) were added to dissolve the formazan crystal, and absorbance was measured at 570 nm using an ELISA reader with Infinite 200 Pro NanoQuant (TECAN). Apoptotic cells were further visualized using a modified TdT-mediated dUTP Nick End Labeling (TUNEL) assay with a DeadEnd Fluorometric TUNEL system (Promega) according to the manufacturer’s protocol.

### Immunofluorescence

Transfected cells were grown on 0.1% gelatin-coated glass chip in 24-well plates. Cells were fixed in 4% paraformaldehyde 24 h after initial plating, permeabilized with 2% NP-40, then blocked and incubated overnight with 10% FBS in PBS containing primary antibodies. Primary antibodies were visualized with Alexa-350 (anti-mouse from Life Technologies), Alexa-488 (anti-rabbit, anti-mouse, and anti-goat from Invitrogen) and Alexa-594 (anti-rabbit, anti-mouse, and anti-rat from Invitrogen) secondary antibodies. Nuclei were visualized with 2 μg/ml Hoechst. Slides were mounted with Dako Fluorescent Mounting Medium (Dako) or UltraCruz Hard-set Mounting Medium containing 1.5 μg/ml DAPI (sc-359850, Santa Cruz Biotechnology) and visualized by fluorescence microscopy (Axioplan2, Zeiss; Olympus IX71, Olympus).

### Image processing

General image processing and final image preparations for publication were performed by using Adobe Photoshop 7.0 (Adobe Systems Inc.).

### Statistical analysis

Statistical analysis was performed with GraphPad Prism 6 statistic software program. Intergroup differences were appropriately assessed using Student *t*-test. For comparison of several groups, one-way analysis of variance (ANOVA) was used followed by post hoc Tukey’s test. All quantitative data are presented as mean ± s.e.m.; P values of less than 0.05 were considered statistically significant. *P < 0.05, **P < 0.01, ***P < 0.001.

## Additional Information

**How to cite this article**: Song, H. *et al*. Stress-induced nuclear translocation of CDK5 suppresses neuronal death by downregulating ERK activation via VRK3 phosphorylation. *Sci. Rep.* **6**, 28634; doi: 10.1038/srep28634 (2016).

## Supplementary Material

Supplementary Information
