# Supplementary material for: Stress-induced nuclear translocation of CDK5 suppresses neuronal death by downregulating ERK activation via VRK3 phosphorylation
Source: Sci Rep. 2016 Jun 27;6:28634. doi: 10.1038/srep28634 (PMC4922050; doi:10.1038/srep28634)
Supplement: Supplementary Information [file srep28634-s1.pdf]

Stress-induced nuclear translocation of CDK5 suppresses neuronal death by downregulating ERK activation via VRK3 phosphorylation.

Haengjin Song, Wanil Kim, Jung-Hyun Choi, Sung-Hoon Kim, Dohyun Lee, Choon-Ho Park, Sangjune Kim, Do-Yeon Kim and Kyong-Tai Kim

SUPPLEMENTARY DATA

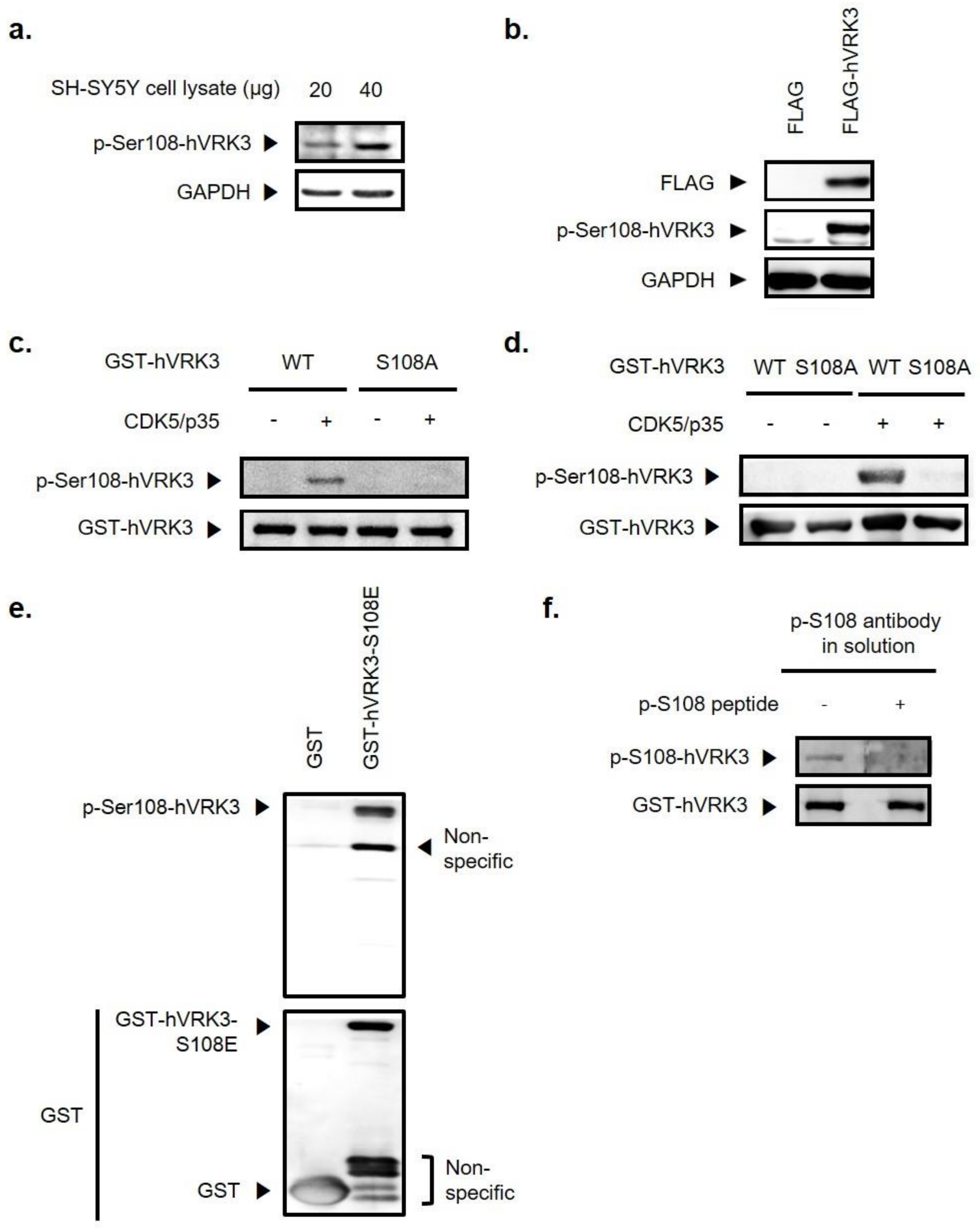

Supplementary Fig. S1. Specific reactivity of purified anti-hVRK3-phospho-S108 antibody. (**a, b**) Detection of phosphorylated VRK3 in SH-SY5Y cell lysates with α-hVRK3-phospho-S108 (pS108-VRK3) antibody raised in either rabbits (a) or rats (b). (**c, d**) CDK5-mediated phosphorylation of recombinant GST-VRK3 at S108 with pS108-VRK3 antibody raised in either rabbits (c) or rats (d). (**e**) Detection of phospho-mimetic mutant of VRK3 (S108E) with pS108-VRK3 antibody raised in rats. (**f**) Specificity of the antibody for pS108-VRK3 antibody raised in rabbits in a peptide competition assay.

Supplementary Table S1. Clinicopathological details of subjects used for Western blotting.

Profile and Clinical data of AD and PD patients and control cases for Western blotting

Diagnosis	NBB no	Age(yr)	Sex	Braak	Amyloid	Braaklb	PMD(hours)
Control	12-070	79	M	2			05:45
	09-300	71	V	1	A		07:10
	12-049	70	V	2	A		07:35
	12-059	78	V	2	A		04:35
AD	08-107	77	M	4	C		04:05
	09-185	70	M	4	C		04:00
	12-022	79	M	4	C		04:05
	07-315	71	M	5	B		05:25
PD	11-117	78	M			4	06:15
	04-108	73	M	1	A	5	05:35
	09-207	67	V	1	B	6	07:40
	09-235	70	M	1	B	5	05:15

NBB no, Netherlands Brain Bank number; Braak: braak stage based on amyloid-beta(0-2: non-demented AD, 3-6: mild to severe)

Amyloid, a type of amyloid; Braaklb, braak stage based on lewy body (0-2: non-demented PD, 3-6: mild to severe);

PMD, post-moterm delay; Control, non-demented control; AD, Alzheimer's disease; PD, Parkinson's disease; M, male.
